# Development of a pain self-management intervention framework for people with spinal cord injury

**DOI:** 10.4102/phcfm.v15i1.4039

**Published:** 2023-10-26

**Authors:** Mokgadi K. Mashola, Elzette Korkie, Diphale J. Mothabeng

**Affiliations:** 1 Department of Physiotherapy, Faculty of Health Sciences, University of the Witwatersrand, Johannesburg, South Africa; 2 Department of Physiotherapy, Faculty of Health Sciences, University of Pretoria, Pretoria, South Africa

**Keywords:** modified e-Delphi, pain, self-management, spinal cord injury, treatment

## Abstract

**Background:**

Pain is the most common reason for medical visits to primary health care practitioners. Pain self-management interventions are encouraged and there is no known self-management intervention framework available that clinicians and people with spinal cord injury (PWSCI) can use to guide treatment selection.

**Aim:**

This study aimed to develop a pain self-management intervention framework for PWSCI.

**Setting:**

Online and facilitated in Gauteng, South Africa.

**Methods:**

A three-round modified e-Delphi method was used to reach an 80% consensus among a 21-expert panel. Fifty-nine interventions were distributed via REDCap and a final online audio meeting was held to either include or exclude interventions in the final framework. SPSS v27 was used to analyse descriptive data and content analysis was used for qualitative responses.

**Results:**

The final developed pain self-management framework consists of 56 interventions and includes interventions from multiple health professions to encompass medical, psychological, therapeutic and social interventions. Interventions are also specified for nociceptive and/or neuropathic pain and grouped according to the biopsychosocial model.

**Conclusion:**

The interprofessional framework may be used as a guideline for PWSCI to alleviate pain, as well as assist health professionals in clinical decision-making, by providing them with the freedom to choose acceptable and adequate interventions that may be appropriate to treat the affected individual’s pain.

**Contribution:**

Pain management is a basic need at the primary healthcare level and PWSCI need access to the broad range of interventions available to manage their pain. The framework highlights the variety of appropriate interventions to guide both health professionals and PWSCI with pain relief options.

## Introduction

A spinal cord injury (SCI) results in partial or complete loss of motor and/or sensory functions below the level of injury and results in common secondary health conditions (SHCs), including pain, pressure ulcers as well as urinary and bowel complications.^1,2^ Secondary health conditions are debilitating and worsen the experience of disability for people with spinal cord injury (PWSCI) by negatively influencing their dignity and independence.^3^ The SHCs also cause PWSCI to consult more with health professionals and cause multiple readmissions to the hospital.^4,5^ Approximately half to two-thirds of PWSCI experience pain, which is an unpleasant sensory and emotional experience that is associated with either actual or potential tissue damage.^6,7^ Pain often commences within the initial 6 months after SCI, with the possibility of being aggravated over time.^8^ The experience and perception of pain may be intense and reported as severe to extreme, negatively affecting health satisfaction and interfering with activities of daily living.^9,10^ The multifactorial pattern of pain ultimately affects how patients react to pain and respond to pain management. In addition, the different underlying mechanisms of pain further challenge pain management.^11^ For example, pain management can be further complicated by a variety of emotional, behavioural and social factors that can negatively affect the experience of pain.^12^ The severity of pain is influenced by various factors such as genetics, comorbidities, current psychological state, prior experience of pain and socioeconomic circumstances,^13^ and treatment is rarely aimed at all associated factors of pain, resulting in ineffective treatment strategies.^14^ Despite the challenges, management of pain is essential, and without intervention, PWSCI may experience additional losses in function and community mobility.^15^

Pain management interventions following SCI include task modifications, therapeutic treatments, and pharmacological and surgical options. Therapeutic interventions such as physiotherapy are recommended as first-line treatment^16^ with a wide range of therapeutic interventions such as thermotherapy, electrotherapy, massage and exercises.^17^ Despite recommendations, pharmacological treatment of pain remains the mainstream choice of pain management in the spinal cord population. However, PWSCI still report that significant pain relief is difficult to achieve, with pharmacological treatments providing very minimal pain relief.^18^ Corticosteroid injections and surgical interventions (such as rotator cuff repairs in the shoulder) are only considered as a last resort to manage severe pain. Surgical intervention is often unsuccessful in PWSCI because of the extended 6-week period of rest required after surgery.^14^ The strict protocols post-surgery hinder independence in wheelchair use^19^ and create a high risk of developing potential SHCs such as pressure ulcers when on bed rest.

Pain is the most common reason for medical visits to primary health care practitioners,^20^ and frequent visits for pain-relieving interventions may not be logistically and financially feasible, especially for affected individuals from low-income households.^21^ Coupled with findings that self-management tasks and skills incorporated into daily life reduce SHCs, it is unsurprising that self-management programmes are steadily being more common.^22,23^ Furthermore, literature has highlighted the need for increased self-management efforts for PWSCI to reduce overall SHCs.^24^ Self-management is defined as the ‘individual’s ability to manage symptoms, treatment, physical and psychosocial consequence and lifestyle changes inherent in living with a chronic condition (p. 178).’^25^ Self-management interventions may refer to a single or combination of approaches that PWSCI can learn (or be taught by any health professional) to enable them with the skills to reduce the impact of the pain on their everyday life.^26^ Behaviour therapy, relaxation techniques, stretching and exercises are among the wide variety of self-management techniques identified to relieve pain.^27^ Self-management also includes the ability of affected individuals to monitor their pain condition and their behavioural, cognitive and emotional responses.^28^ Self-management includes problem-solving, decision-making, using resources, communicating with health professionals and taking action when needed.^29^ People with SCI must have self-management programmes targeted at all three categories because of the complexity of SCI, which often present with multiple chronic conditions.^30^

Self-management of chronic pain requires that affected individuals distinguish themselves from the pain they experience so that they do not define themselves by their pain.^31^ Barriers to self-management include the overwhelming effort to manage pain, unsupportive family members or health professionals, and limited problem-solving abilities.^31,32^ There is a need for increased self-management efforts by both healthcare providers and PWSCI to reduce SHCs in PWSCI,^33^ and this study aimed to develop a guided pain self-management intervention framework from the perspective of HCPs working with PWSCI.

## Research methods and design

### Study design

This study utilised a qualitative approach and a Delphi design to obtain the most reliable consensus from a group of experts by using surveys to receive controlled feedback on their opinions, especially when there is little or no definitive evidence on the subject.^34,35,36^ A Delphi design involves an iterative process that is useful in gathering subjective information from experts working in the field of interest and there are variations on the classic Delphi method.^37^ The classic Delphi is modified by presenting the experts with a predetermined list of interventions that may be used to manage the pain instead of the experts determining the list, hence the term ‘modified Delphi’. The predetermined list comprises interventions to manage pain and not specifically self-management interventions, as the experts needed to select the interventions that would be appropriate for self-management. The predetermined list of interventions was derived from phase 2 of a mixed-method study by the authors and published literature. Phase 2 explored the experience of pain by PWSCI and the coping strategies they used to manage their pain, following guidance from the first quantitative phase. The first phase found that 85% of PWSCI reported pain with a severity of 6.7/10.^38^ This study is the third phase of this mixed-method study.^39^ The approach is referred to as a ‘modified e-Delphi’ design in this study because all three rounds of the modified Delphi were conducted online.^37^ The modified e-Delphi study was conducted and informed by a predefined and published protocol^39^ and is reported as per the Conducting and Reporting Delphi Studies (CREDES) recommendations.^40^

### Setting

This study was conducted online: REDCap for the first two rounds and Microsoft Teams for the third and final round.

### Study population and sampling strategy

Healthcare professional experts were identified from a variety of different professional backgrounds in the field of SCI and/or pain management in academia and clinical practice. The experts were identified from their global contribution to the following associations: International Spinal Cord Society, Africa Spinal Cord Injury Network, Southern African Spinal Cord Association, and PainSA. Experts in academia needed to be in a possession of a master’s degree and experts in clinical practice had to have at least 10 years of experience in their respective fields. Sixty-four experts were purposefully sampled and invited via email to participate in the study. A detailed information sheet explaining the study aims, process and informed consent was emailed to the experts before participating in the study.

### Data collection procedure

**FIGURE 1:**
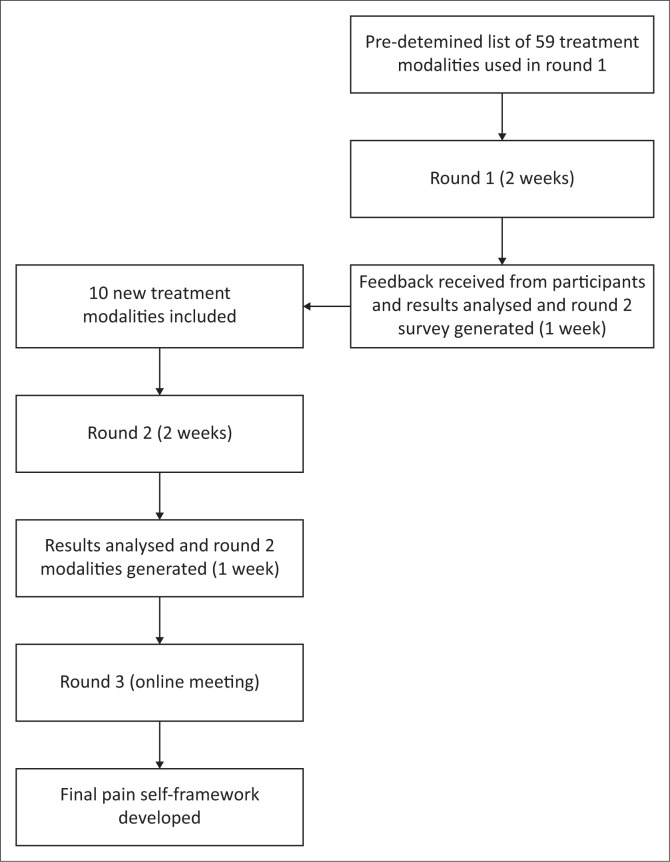
The procedure of this modified e-Delphi study.

Figure 1 shows the procedure of this modified e-Delphi study. A pilot study with the same procedure as planned for the main study was conducted, as guided by Clibbens et al.^41^ Two experts meeting the inclusion criteria were included in the pilot study (one in the SCI field and the other in the pain management field). The pilot study was conducted over 1 week; feedback was received and collated and modifications were implemented before commencing with the main study. Results from the pilot study were not included in the main results as changes were made, and the two experts who participated in the pilot study were not included in the main study, but remained experts for the round two pilot study.

#### Round one

REDCap was used to construct and distribute the predetermined interventions. Participants were given 2 weeks to complete the survey and those who did not respond by the end of the 2 weeks were removed from the study. Reminders were sent at the end of the first week and 2 days before the deadline. The participants were given the option to select ‘agree’, ‘disagree’ or ‘do not know’ for each intervention as guided by Eubank et al.^34^ Participants were required to give reasons for their selection in the text box provided and also had the option of adding comments and extra interventions that were not on the predetermined list. The third week was allocated for data analysis. The items were analysed descriptively using SPSS v27 to determine the 80% consensus^34^ and content analysis was used to analyse the data from the free text boxes. Interventions that did not receive consensus were redistributed in round two without giving participants feedback on how the items were scored to maintain impartiality.

#### Round two

Feedback from experts in the comment sections recommended that the researchers consider giving more information over and above the descriptions provided for each intervention. Therefore, one moderate-to-high level of evidence article was included per intervention in round two. Medline (PubMed) was used to search for each intervention that was redistributed in round two. In this round, participants had to either ‘agree’ or ‘disagree’ only; thus the ‘do not know’ option was removed to receive firm decisions from the participants. Similar to the previous round, the participants were also required to give reasons for their selection in the text box provided. A pilot study was also conducted for round two by the same experts who were involved in the initial pilot study. Comments and feedback from this pilot study were collated and changes to the round two survey were carried out. The pilot study results were also not included in the main study because some changes were required for the main study and the experts involved in the pilot study did not participate in round two. The survey for round two was also distributed via REDCap and conducted over 2 weeks. Reminders were sent as per round one and non-responders were removed from the study. The same steps as in round one were followed to analyse the data.

#### Round three

The aim of round three was to discuss the interventions that did not reach 80% agreement to include or exclude from the final framework. Participants who responded in round two were sent a *‘Doodle poll’* link to select their preferred time slot over 14 days to conduct round three via Microsoft Teams, and were not anonymous. The date and time when most of the experts across different professions were available were chosen as the day of the meeting. Interventions that did not reach the 80% consensus in round two were redistributed in round three and there was expert interaction to exchange important information and reasons for disagreements were clarified. Previous interventions that met the 80% consensus in round two but received objections in round three were discussed and removed following unanimous decisions to do so. Only the interventions that reached 80% consensus at the end of this round were added to the final framework. The consensus was decided by a vote of hands, where the participants typed a ‘thumbs up or thumbs down’ in the chat and the lead author counted the votes for each intervention. The final list of interventions was grouped for neuropathic and/or nociceptive pain and also according to the biological, psychological and social components of the biopsychosocial model of care.

### Data analysis

The SPSS v27 was used to descriptively analyse the participants’ demographic information, as well as the items to determine consensus. Data were reported in frequencies, percentages, means and standard deviations. Qualitative data were analysed using content analysis as guided by Erlingsson and Brysiewicz.^42^ Apart from maintaining consensus in all the rounds, rigour was also controlled in the last round by giving the experts the opportunity to revise the findings of the two rounds, using consensus, to determine which responses were appropriate.^43^ Furthermore, the authors kept a methodological journal during the study to document the major decisions and the interventions that sparked debates.

### Ethical considerations

This modified e-Delphi study is the third phase of the mixed-method study that is registered with the South African National Health Research Database (reference GP201806005) and received ethical approval from the Faculty of Health Sciences Research Ethics Committee of the University of the Pretoria, South Africa (approval number 125/2018). Written informed consent was obtained from all the participants in this study, including consent for the third round to be audio-recorded. Furthermore, participants’ names are not used to ensure confidentiality.

## Results

### Participants’ demographic information

Twenty-two participants from South Africa, Sweden and Canada accepted the invitation and 21 participants completed the survey and were included in the study. The mean age of the participants was 42.55 years (s.d. = 10.53) and the mean year practicing in their professions in their highest qualification was 11.09 years (s.d. = 10.47). The majority of the participants had a master’s degree (*n* = 10, 45.5%), practiced in the clinical setting (*n* = 12, 54.6%) and were mainly physiotherapists (*n* = 8, 36.4%) as shown in Table 1.

**TABLE 1:** Participants’ information (*n* = 21).

Description	*n*	%
**Gender**		
Male	5	23.8
Female	16	76.2
**Profession**		
Dietitian	1	4.8
Medical doctor	4	19.0
Occupational therapist	4	19.0
Peer counsellor	2	9.5
Pharmacist	1	4.8
Physiotherapist	7	33.3
Psychologist	1	4.8
Registered nurse	1	4.8
**Country**		
Canada	1	4.8
Sweden	2	9.1
South Africa	18	81.8
**Highest qualification**		
Matric	1	4.8
Degree in Management Nursing	1	4.8
Bachelor of Science	5	23.8
Master of Science	8	38.1
Master of Medicine	1	4.8
Bachelor of Medicine and Bachelor of Surgery	3	14.3
Doctor of Philosophy	2	9.5
**Work setting**		
Academia	6	28.6
Clinical	13	61.9
Corporate	2	9.5
Government	1	4.8
**Field of expertise**		
Spinal cord injury	13	61.9
Pain management (including emotional well-being)	4	19.0
Spinal cord injury and pain management	3	14.3
Other (peer counselling)	1	4.8
**Number of publications**		
Zero	10	47.6
1–5	8	38.1
> 5	3	14.3

### e-Delphi information

#### Round one

Table 2 illustrates the descriptive statistics of the predetermined items, with the first column specifying where the interventions were sourced from. Twenty-one participants completed round one (95.5% response rate) and one participant did not complete the survey. The consensus was achieved with 80% agreement for 34 interventions out of the original 59. Twenty-five interventions, therefore, did not achieve consensus in this round.

**TABLE 2:** Round one descriptive statistics of the items (59 interventions).

Source	Interventions	Percentage of agreement (%)	Consensus achieved
Phase 2	Tourniquet application	9.5	No
	Pain neuroscience education	90.5	Yes
	Lifestyle modifications	100.0	Yes
	Surgery	14.3	No
	Prescribed medication: Acetaminophens	71.4	No
	Prescribed medication: Opioids	76.2	No
	Prescribed medication: Non-steroidal anti-inflammatories	90.5	Yes
	Prescribed medication: Anticonvulsants	57.1	No
	Prescribed medication: Tricyclic antidepressants	81.0	Yes
	Prescribed medication: Serotonin-selective reuptake inhibitors (SSRI)	52.4	No
	Prescribed medication: Serotonin-norepinephrine reuptake inhibitors (SNRI)	52.4	No
	Relaxation techniques	95.2	Yes
	Modified yoga	95.2	Yes
	Position changes to relieve pain	95.2	Yes
	Exercises: gravity-eliminated physiological joint movements of partially innervated areas	71.4	No
	Exercises: physiological joint movements against gravity (innervated areas)	76.2	No
	Exercises: resisted strength training (innervated areas)	85.7	Yes
	Weight-bearing exercises	85.7	Yes
	Conventional massage	81.0	Yes
	Thermotherapy: heat	85.7	Yes
Literature search	Treatment of aggravating secondary health conditions: Pressure ulcers	85.7	Yes
	Treatment of aggravating secondary health conditions: Urinary tract infections	90.5	Yes
	Treatment of aggravating secondary health conditions: Constipation	95.2	Yes
	Prescribed medication: Anxiolytics	47.6	No
	Prescribed/over-the-counter medication: Topical agents	76.2	No
	Prescribed medical cannabis where legal	66.7	No
	Recreational cannabis	14.3	No
	Cognitive behavioural therapy	95.2	Yes
	Mindfulness	95.2	Yes
	Psychological control techniques	95.2	Yes
	Resilience coping strategies	81.0	Yes
	Adjusted sleep and rest	90.5	Yes
	Graded exposure therapy	90.5	Yes
	Psychoeducational management	90.5	Yes
	Self-hypnosis	33.3	No
	Formal peer support systems	85.7	Yes
	Informal support systems	90.5	Yes
	Role of the family in care	85.7	Yes
	Help-seeking behaviour	90.5	Yes
	Spirituality	85.7	Yes
	Thai massage	28.6	No
	Acupuncture	38.1	No
	Group exercises	90.5	Yes
	Participation in meaningful leisure activities	95.2	Yes
	Basic body awareness training	71.4	No
	Active stretches of innervated areas	90.5	Yes
	Dynamic stretches of innervated areas	90.5	Yes
	Passive stretches of non-innervated areas	85.7	Yes
	Auto-assisted joint mobilisations: Maitland	38.1	No
	Auto-assisted joint mobilisations: Mulligan	33.3	No
	Exercises: aerobic cardiovascular exercises	90.5	Yes
	Exercises: core exercises of partially and fully innervated areas	95.2	Yes
	Soft tissue mobilisation	71.4	No
	Hydrotherapy	95.2	Yes
	Thermotherapy: Cold	52.4	No
	Electrotherapy in innervated areas	52.4	No
	Electrotherapy in non-innervated areas	23.8	No
	Transcutaneous electrical nerve stimulation (TENS) in both innervated and non-innervated areas	71.4	No
	Dry needling	61.9	No

Tourniquet application, surgery and recreational cannabis had the majority of disagreements to be included in the final framework; however, the disagreements did not reach 80% consensus (61.9%, 47.6% and 61.9%, respectively). Most of the participants did not know whether to include self-hypnosis (47.6%), Thai massage (52.4%), Maitland and Mulligan joint mobilisations (42.9% and 57.1%, respectively) and electrotherapy to non-innervated areas (38.1%). Although not in the majority, pharmacological interventions saw many participants not knowing whether to include the specific drug groups, namely, anticonvulsants (42.9%), serotonin-selective reuptake inhibitors (SSRI) (42.9%) and serotonin-norepinephrine reuptake inhibitors (SNRI) (47.6%), antidepressants, as well as anxiolytics (42.9%).

Following the inductive thematic analysis of round 1, three themes were identified. The qualitative results support the quantitative results as it was found that most ‘do not know’ responses were because of concerns about medications that need to be prescribed appropriately, interventions falling outside of one’s scope, and more information necessary before decisions could be made about including or excluding interventions from the framework. The thematic analysis guided the researchers to include the literature in the second round.

#### Theme one: Medication should be appropriately prescribed

There was a general concern with medication dependency and organ function with prolonged use, and participants reported that there must be the correct use of the medication with consideration of side effects:

‘Opioids are addictive and may lead to poor quality of life in the long run.’ (Participant 16, 46 years, MOccTher)

‘Medication is useful when used appropriately and under the guidance of a clinician or medical practitioner.’ (Participant 4, 47 years, MSc Occupational Therapy)

#### Theme two: Beyond the scope of practice

There was a general unfamiliarity with certain interventions that fell outside one’s scope of practice:

‘Physiotherapists do not prescribe drugs.’ (Participant 5, 59 years, PhD Physio)

‘I’m not aware of this for pain management.’ (Participant 8, 28 years, BSc OT)

#### Theme three: There is a need for more information on the interventions

On some interventions that were not as popular as others (e.g. body awareness training), participants commented they needed to familiarise themselves with some of the interventions:

‘I would need to do more research in this regard.’ (Participant 2, 29 years, BSc Physio)

‘I am not familiar with this technique.’ (Participant 1, 34 years, MBCHB)

Ten more pain self-management interventions were added to round two following the content analysis of suggestions from the participants and are shown in Table 3.

**TABLE 3:** Round two descriptive statistics of the items.

Intervention	Percentage of agreement (%)	Consensus achieved	Reference of the article used	Type of study	LOE
Tourniquet application	25.0	No	Navein et al.	Commentary review	V
Surgery	56.3	No	Rothemeyer et al.	Literature review	V
Prescribed medication: Acetaminophens	100.0	Yes	Pickering et al.	double-blind cross-over study	II
Prescribed medication: Opioids	87.5	Yes	Teasell et al.	Systematic review and meta analysis	I
Prescribed medication: Anticonvulsants	100.0	Yes	Davari et al.	Systematic review and meta analysis	I
Prescribed medication: Serotonin-selective reuptake inhibitors (SSRI)	87.5	Yes	Baltenberger et al.	Systematic review and meta analysis	I
Prescribed medication: Serotonin-norepinephrine reuptake inhibitors (SNRI)	100.0	Yes			
Prescribed medication: Anxiolytics	81.3	Yes	Wright	Narrative review	V
Prescribed/over-the-counter medication: Topical agents	87.5	Yes	Neuropathic pain: Jackson MSK pain: Stanos	Narrative review Special review	V V
Prescribed medical cannabis where legal	87.5	Yes	Stillman et al.	Cross-sectional	III
Recreational cannabis	31.3	No	Nabata et al.	Systematic review and meta analysis	I
Self-hypnosis	75.0	No	Jensen et al.	RCT	I
Thai massage	81.3	Yes	Netchanok et al.	Systematic review	I
Acupuncture	62.5	No	Heo et al.	Systematic review and meta analysis	I
Basic body awareness training	87.5	Yes	Lundwall et al.	Qualitative study	III
Auto-assisted joint mobilisations: Maitland	81.3	Yes	Ali et al.	Repeated measure design	III
Auto-assisted joint mobilisations: Mulligan	75.0	No	Hing et al.	Systematic review	I
Exercises: Gravity-eliminated physiological joint movements (partially innervated areas)	93.8	Yes	Geneen et al.	Systematic review	I
Exercises: physiological joint movements against gravity (innervated areas)	100.0	Yes			
Soft tissue mobilisation	93.8	Yes	Costello et al.	RCT	I
Thermotherapy: Cold	56.3	No	De Alencar Caldas et al.	RCT	I
Electrotherapy in innervated areas	81.3	Yes	Fuentes et al.	Systematic review and meta analysis	I
Electrotherapy in non-innervated areas	62.5	No	Anju et al.	Systematic review	I
Transcutaneous electrical nerve stimulation (TENS) in both innervated and non-innervated areas	81.3	Yes	Celik et al.	RCT	I
Dry needling	75.0	No	Kietrys et al.	Systematic review and meta analysis	I
**Suggested interventions**					
Prescribed medication: Muscle relaxants for spasms	100.0	Yes	Teasell et al.	Systematic review and meta analysis	I
Neurofeedback therapy	93.8	Yes	Patel et al.	Systematic review and meta analysis	I
Somatic experiencing	68.8	No	Andersen et al.	RCT	II
Wim Hof breathing technique	62.5	No	Muzik et al.	Case study	V
Neural tissue mobilisations	87.5	Yes	Su and Lim	Systematic review and meta analysis	I
Seating and ergonomics	100.0	Yes	Burns et al.	Non-randomised experimental	II
Dynamic taping technique	75.0	No	Alahmari et al.	RCT	I
Tension trauma release exercises	56.3	No	Lynning et al.	Non-randomised experimental pilot	III
Education on correct positioning in bed and wheelchair	100.0	Yes	Burns et al.	Non-randomised experimental	II
Treatment of other aggravating secondary health complications: Contractures, postural abnormalities, tendon, and neural shortening, systemic infections, ingrown toenails	93.8	Yes	Namdari et al.	Case series	V

LOE, level of evidence; RCT, randomised control trial.

References used in Round 2 (Table 3)

Note: Please see the full reference list of the article, Mashola MK, Korkie E, Mothabeng DJ. Development of a pain self-management intervention framework for people with spinal cord injury, Afr J Prm Health Care Fam Med. 2023;15(1), a4039. https://doi.org/10.4102/phcfm.v15i1.4039, for more information.

### Round two

Sixteen participants completed round two (76.2% response rate) and five participants did not complete the survey. The consensus was achieved with 80% agreement for 22 out of the 35 total interventions. Thirteen interventions, therefore, did not achieve consensus in this round as shown in Table 3^44,45,46,47,48,49,50,51,52,53,54,55,56,57,58,59,60,61,62,63,64,65,66,67,68,69,70,71,72,73,74,75^. Six of the 10 new interventions that were included in round two reached a consensus. Of the initial interventions, tourniquet application, surgery and recreational cannabis still showed disagreement to be included (75%, 43.8% and 68.8%, respectively) and 10 interventions (40%) from the original 25-item list did not reach consensus. Table 3 also depicts the references of the articles used in this round and the respective level of evidence (LOE) as guided by the Johns Hopkins Nursing Evidence-Based Practice.^76^

Two themes were identified following the inductive thematic analysis of round two. Concerns were raised about side effects and the availability of the equipment that would be necessary for some of the interventions.

#### Theme one: Adverse effects are too risky

Although consensus was not reached to exclude some interventions from the framework, most of the participants felt strongly about interventions they deemed unsafe and untested:

‘This is such an esoteric technique that requires training that I think it is totally impractical to be accepted as a mainstream treatment for post-SCI pain.’ (Participant 16, 46 years, MOccTher)

‘Sounds as though this method is cost-effective and easy to use but research does not prove the effect of this therapy approach.’ (Participant 1, 34 years, MBCHB)

‘This technique is high risk for patients with impaired sensation and are already potentially at high risk for circulatory disorders.’ (Participant 11, 53 years, MMed(Ortho))

#### Theme two: Availability of resources

Participants expressed concerns regarding the availability of some interventions, especially for patients in low-resourced areas:

‘This modality is not widely available in the South African setting and is not funded. Although I feel it would be helpful in reality very few patients could receive this.’ (Participant 16, 46 years, MOccTher)

### Round three

Thirteen interventions that did not reach consensus in round two were redistributed and discussed in round three via an audio conference meeting on Microsoft Teams. Fourteen participants responded to the ‘Doodle Poll’ and nine participants were available at one time slot and subsequently accepted the meeting invite. The nine participants included four physiotherapists, three occupational therapists and two medical doctors. One physiotherapist did not join the meeting and a final eight experts (all from the medical fratenity) participated in round three (50% response rate from round two). The consensus was achieved with 80% agreement for all 13 interventions that did not meet the consensus in round two to be excluded from the final framework as shown in Table 4. The meeting was conducted in English and was recorded and transcribed.

**TABLE 4:** Round three descriptive statistics of the items.

Interventions	Percentage of the agreement to exclude item (%)	Consensus achieved
Tourniquet application	100.0	Yes
Surgery	100.0	Yes
Recreational cannabis	100.0	Yes
Self-hypnosis	100.0	Yes
Acupuncture	100.0	Yes
Auto-assisted joint mobilisations: Maitland	100.0	Yes
Thermotherapy: cold	87.5	Yes
Electrotherapy in non-innervated areas	100.0	Yes
Dry needling	100.0	Yes
Somatic experiencing	100.0	Yes
Wim Hof breathing technique	100.0	Yes
Dynamic taping technique	100.0	Yes
Tension trauma release exercises	100.0	Yes

Table 5 depicts the consensus to specify the type of pain for each item. Following robust discussion, Thai massage and auto-assisted Maitland joint mobilisations, which both received 81.3% agreement in round two to include in the framework, were excluded following a unanimous decision. The reasons cited by the experts included that masseuses providing Thai massage were not medically trained and that Maitland techniques often need a therapist to effectively provide the necessary pain relief.

**TABLE 5:** Developed guided pain self-management intervention framework (56 interventions).

Subcategory of interventions	Interventions	Type of pain	
		Nociceptive pain	Neuropathic pain
Medical	Pain neuroscience education (PNE)	✓	✓
	Treatment of aggravating secondary health complications: pressure ulcers	✓	✓
	Treatment of aggravating secondary health complications: urinary tract infections	✓	✓
	Treatment of aggravating secondary health complications: constipation	✓	✓
	Treatment of other aggravating secondary health complications: contractures, postural abnormalities, tendon, and neural shortening, systemic infections, ingrown toenails	✓	✓
	Education on correct positioning in bed and wheelchair	✓	✓
Pharmacological	Prescribed medication: acetaminophen	✓	
	Prescribed medication: opioids	✓	✓
	Prescribed medication: NSAIDs	✓	
	Prescribed medication: anticonvulsants	-	✓
	Prescribed medication: antidepressants (tricyclic)	-	✓
	Prescribed medication: serotonin-selective reuptake inhibitors (SSRI)	-	✓
	Prescribed medication: serotonin-norepinephrine reuptake inhibitors (SNRI)	-	✓
	Prescribed medication: anxiolytics	✓	✓
	Prescribed medication: topical agents	✓	✓
	Prescribed medication: cannabis	✓	✓
	Prescribed medication: muscle relaxants	✓	✓
Psychotherapeutic	Cognitive behavioural therapy (CBT)	✓	✓
	Mindfulness	✓	✓
	Psychological control techniques	✓	✓
	Resilience coping strategies	✓	✓
	Adjusted sleeping and resting	✓	✓
	Graded exposure therapy	✓	✓
	Relaxation techniques	✓	✓
	Modified yoga	✓	✓
	Psychoeducational management	✓	✓
Socioenvironmental	Lifestyle modifications	✓	✓
	Formal peer support systems	✓	✓
	Informal support systems	✓	✓
	Role of the family in care	✓	✓
	Help-seeking behaviour	✓	✓
	Spirituality	✓	✓
	Position changes to relieve pain	✓	✓
	Group exercises	✓	✓
	Participation in meaningful leisure-time activity	✓	✓
	Basic body awareness training (BBAT)	✓	✓
Physiotherapeutic	Stretches: active stretches of innervated areas	✓	✓
	Stretches: dynamic stretches of innervated areas	✓	✓
	Stretches: passive stretches of non-innervated areas	✓	✓
	Exercises: gravity-eliminated physiological joint movements of partially innervated areas	✓	✓
	Exercises: physiological joint movements against gravity of innervated areas	✓	✓
	Exercises: resisted strength training of innervated areas	✓	✓
	Exercises: aerobic cardiovascular exercises	✓	✓
	Exercises: core exercises of partially and fully innervated areas	✓	✓
	Weight-bearing exercises	✓	✓
	Soft tissue mobilisation	✓	✓
	Conventional massage therapy	✓	✓
	Hydrotherapy	✓	✓
	Thermotherapy: heat (with caution on spines)	✓	✓
	Electrotherapy: innervated areas	✓	✓
	Transcutaneous electrical nerve stimulation (TENS)	-	✓
	Neurofeedback therapy	✓	✓
	Seating and ergonomics	✓	✓
	Neural tissue mobilisation	✓	✓

NSAIDs, Non-steroidal anti-inflammatory drugs.

The general agreement by the participants was that some of the interventions need to be adequately taught to PWSCI by qualified health professionals where applicable. Furthermore, participants emphasised the following interventions:

- All prescribed medication: The source of pain must be considered and the prescription must be appropriate for optimum pain relief and avoidance of dependency.
- Psychoeducational management: Emphasis was placed more on neuropathic pain than nociceptive pain.
- Active stretches of innervated areas: Emphasis was placed more on nociceptive pain.
- Transcutaneous electrical nerve stimulation (TENS): To be applied with caution and individuals must be taught the correct parameters before application.

**FIGURE 2:**
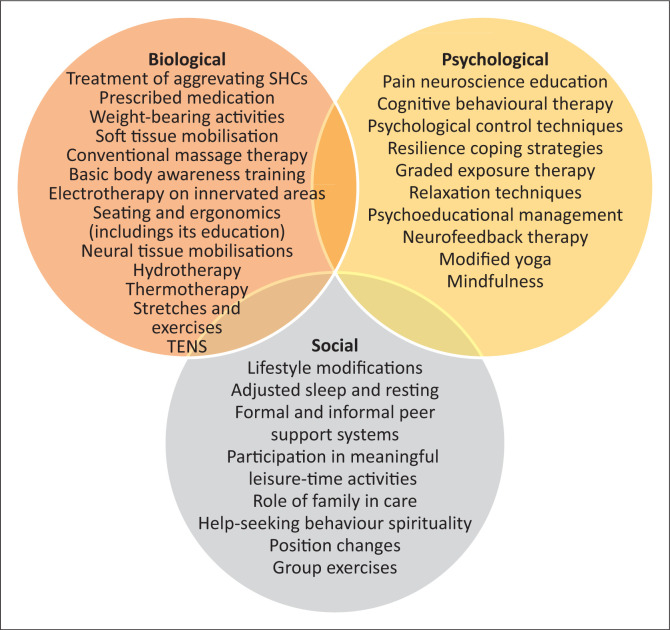
The biopsychosocial aspects of the developed guided pain self-management intervention framework.

Figure 2 illustrates the final guided pain self-management intervention framework as per the biopsychosocial model.

## Discussion

This study derived an expert consensus list of interventions to include in a guided pain self-management intervention framework for PWSCI. The overall response rate was 73.9% across the three rounds, which is slightly lower than the 80% overall response rate from studies reviewed by Gargon et al.^85^ Literature has found that a higher number of items tend to result in lower response rates in subsequent rounds and vigorous strategies, such that up to four reminders are necessary for higher response rates.^77,78^

The final framework consists of 56 interventions that are appropriate for nociceptive and/or neuropathic pain and span across the different domains of the biopsychosocial model. Lifestyle modification was the only intervention that received unanimous agreement from experts in the first round, suggesting that the need for individual-based management may be more important than medical, psychological or therapeutic interventions in isolation. Lifestyle changes are person-specific and may include changes to alcohol use, smoking, as well as leisure-time physical activity, and diet among others. Excessive alcohol use is associated with increased pain severity and interference,^79^ while cigarette smoking is found to have both causal and reciprocal effects on pain in the general population^80^ and worsening pain in PWSCI.^81^ Increased leisure-time physical activity as well as physical activity targeted at weight loss is associated with decreased pain levels and improved health satisfaction in PWSCI.^82,83,84^ Lifestyle changes can be achieved by using self-management programmes that focus on behaviour change.^85^

The majority of the psychological interventions reached ≥ 90% agreement including cognitive behavioural therapy (CBT), mindfulness, psychological control techniques and psychological educational management, which are some of the most common self-management interventions.^27,32^ Although included in the final framework, many of the pharmacological interventions only reached consensus in the second round following the inclusion of literature evidence. This is unsurprising as the panel of experts only consisted of one pharmacist and four medical doctors. Tricyclic antidepressants and NSAIDs were the only groups of medications that were agreed on in the first round. Although this may seem as if the experts did not, at first glance, agree for all groups of medications to be part of self-management, likely, tricyclic antidepressants and NSAIDS are still known by the experts to be first-line pharmacological interventions for neuropathic and nociceptive pain, respectively. Although NSAIDs remain appropriate for nociceptive pain, anticonvulsants such as pregabalin are now the first-line medication for neuropathic pain.^85,86^ The content analysis identified that medication should be appropriately prescribed and will only be useful when used as prescribed and only under the guidance of a health professional. Inappropriate medication prescription is a public health concern, especially in vulnerable groups such as the elderly and people with disabilities.^87^ Prescribers should, therefore, take care to not only prescribe appropriately but also address the adverse outcomes of prescribing medication.^88^ Pain medication misuse is common in PWSCI and the risk is greater for those with increased pain severity.^89^ Prescribers need to also consider alternative interventions to reduce the potentially catastrophic consequences of pain medication misuse (such as death from overdosing).

Although the interventions that were eventually excluded did not reach a consensus to be excluded from round one, there was consistent agreement among experts to exclude tourniquet application, surgery, recreational cannabis and electrotherapy in non-innervated areas. There was a major safety concern when PWSCI need to be performing some interventions that were suitable for self-management without supervision. Indeed, maintaining control over one’s care is a facilitator in self-management;^33^ however, techniques need to be performed correctly to not only be effective but also to prevent injury. The inclusion of the tourniquet application was a surprise to many experts, as this is a known intervention to stop blood supply and can be dangerous in individuals with already poor blood circulation and impaired sensation. Tourniquets are only applied during surgery of the limbs and are already known for possible nerve, muscle and skin injury.^90^ One person with SCI used this technique to ease neuropathic pain below the level of injury. A panel discussion by Evans et al.^91^ concluded that blood flow restriction training was safe and effective for improved skeletal muscle strength, when performed by a skilled health professional. However, the potential for pain relief needs further research, including its use as a self-management technique because of the potential adverse effects. The need to achieve pain relief, coupled with other factors such as barriers to accessing health facilities, leads PWSCI to attempt anything, including recreational cannabis.^92^ Although there is some effectiveness shown with the use of cannabis in PWSCI,^92,93^ cannabis use is still understudied, and inclusion of medicinal cannabis in this framework is performed on the condition that the country of use has legalised it and has been prescribed by a health professional. Experts in this study pointed out that some of the interventions are not widely available in the local context. Although some interventions have proven effective in other countries, it may not always be implementable in another country such as South Africa.^94^ This may be because of the various factors such as differences in the communities, attitudes and beliefs, or financial accessibility. The interventions were therefore included on the premise that they would be available for PWSCI in the local context.

By including the ‘do not know’ option in round one, the authors were able to determine that although healthcare professionals work within a multidisciplinary approach in SCI rehabilitation, there is inadequate information known about interprofessional pain management techniques. This may pose a problem where advocacy is needed for a patient regarding adverse effects picked up by another professional. For example, although physiotherapists in South Africa do not prescribe drugs, knowing the effects of the drug assists in knowing what to expect during therapy or when certain self-techniques are prescribed. A medical practitioner would not know how to adequately advise a patient who is not responding well to non-medical interventions. Therefore, appropriate referrals will need to be made for a holistic treatment for PWSCI with pain to improve HCP practice. The onus rests on the HCPs to increase their knowledge of the various interventions available and expertise in managing pain using other techniques within their scope of practice. Although more difficult to establish, an interdisciplinary approach has been shown to increase the effectiveness of pain management programmes^95,96^ and could be a consideration for PWSCI.

### Strengths and limitations

The limitation of a Delphi study is that the expert opinions are often based on either their experiences or biased. However, the results of Delphi studies form an important foundation for decisions that are relevant for clinical practice, especially because clinical guidelines are often grounded on expert opinions and experiences.^40^ This study had a high response rate in the first two rounds, losing only 4.5% of the experts in round one and 23.8% in round two. There was a 50% response rate in round three and this attrition rate may be because of the online meeting being held during working hours. All experts were employed and availability would not be possible for some experts despite the wide range of timelines over the 14 days given to them. Furthermore, experts resided in different countries and had up to 9 h of the time difference, further making some experts unavailable for certain time slots. Hasson et al.^97^ have reported on attrition because of the length of commitment as the rounds progressed. It was initially planned for 7–14 experts^39^ but opted to invite more experts in anticipation of a possible decline in the response rate as the rounds progressed. Although the study commenced with an expert from each profession involved in SCI care, the participants were not evenly distributed across the different professions, resulting in some professions not being represented in the final round because of attrition or unavailability.

Following comments from participants that more information was needed on certain interventions, articles were included on all the interventions and the participants were encouraged to read up on the interventions themselves. Round two was piloted to ensure credibility; however, the authors take into consideration that the inclusion of the literature may have not prevented bias, because the participants were not expected to make their own research before participating in this study. Despite the inclusion of the literature in round two, 40% of the interventions still did not reach a consensus and needed to be discussed in round three, suggesting that those who did not agree in round one did not change their mind in round two. Following the conclusion of the meeting, the experts involved confirmed that the final document was the true representation of the meeting.

Weaknesses of the developed framework include having no items on education that are specifically tailored to motivate towards self-management. It is important to include formalised patient education programmes that will guide the health behaviour changes so that PWSCI may be able to relieve the pain.^98^ There were no items about telerehabilitation, and in light of the coronavirus disease 2019 (COVID-19) pandemic, it may be worthwhile to determine which self-management techniques may be conducted using such technology. Telerehabilitation may be telephone- or internet-based and may be beneficial in not only improving communication with health professionals in the core skills of self-management but also ensuring that prescribed interventions are performed accordingly. Telephone-based counselling can be considered for affected individuals who have to isolate or quarantine for prolonged periods, as this technique has been shown to improve pain management,^99^ coping skills, and reduce depression in people with disabilities.^27^ Notwithstanding the aforementioned limitations, the developed guided pain self-management intervention framework has the potential to improve pain management after SCI.

### Recommendations

Although the evidence does highlight the involvement of some health professions over others in SCI care, it is recommended that future Delphi studies employ specific sampling strategies to ensure that all professions are adequately recruited to prevent loss of representativeness as the rounds progress. It is also recommended that further research be conducted to test the clinical suitability of the interventions included in this framework.

## Conclusion

This study created a list of interventions that were informed by PWSCI and based on the opinions of health professional experts that may be used to manage pain for PWSCI. Some of the interventions could be applied by PWSCI at home following appropriate training on how to perform them independently. The final framework consists of 56 interventions that may be appropriate for nociceptive and/or neuropathic pain as selected by a panel of health professionals, and span across the different domains of the biopsychosocial model. Most of the experts agreed that interventions are only as effective when administered correctly, and thus proper education on how to perform the interventions adequately and safely is imperative. It must be noticed that all the interventions that were agreed on would not necessarily be appropriate for all PWSCI, and the type, area and severity of the pain would need to be thoroughly assessed. Furthermore, the effectiveness of these interventions needs to be established before the implementation of this framework into clinical practice. This is the first framework of self-management interventions developed for the SCI population to the knowledge of the authors. Pain after SCI has been reported to be difficult to manage and the authors envision this framework to play a vital role in assisting health professionals to know the array of interventions appropriate to guide PWSCI towards pain relief. Having a sense of control over one’s care is positively associated with emotional well-being and an improved ability to deal with stress. An individual’s belief in their capabilities can positively impact mood, subsequently reducing the experience of their pain.
